# Retrieval augmented generation in dentistry: potentials, applications, and future directions

**DOI:** 10.3389/fdmed.2025.1760990

**Published:** 2026-02-05

**Authors:** Tahereh Firoozi, Rojin Adabdokht, Hollis Lai

**Affiliations:** Mike Petryk School of Dentistry, Faculty of Medicine and Dentistry, University of Alberta, Edmonton, AB, Canada

**Keywords:** artificial intelligence in dentistry, large language models, natural language processing, retrieval augmented generation, text mining

## Abstract

Retrieval Augmented Generation offers a robust framework for developing reliable and evidence- aligned artificial intelligence in dentistry. By integrating external knowledge sources with the generative capabilities of large language models, RAG addresses key limitations of standalone LLMs, including outdated parametric knowledge, limited domain specificity, and hallucinations. This mini-review outlines the foundations of RAG, its core architectural components, and early dental applications that demonstrate improved factual accuracy and interpretability. Despite promising progress, research remains preliminary, constrained by heterogeneous methods and limited dental corpora. Advancing RAG will require standardized evaluation frameworks, curated knowledge bases, and multimodal resources to support trustworthy AI in dental research and practice.

## Introduction

The application of artificial intelligence in dentistry has evolved from classical machine learning models engineered around hand-crafted features (1) to deep learning systems (2) that learn representations directly from data, and most recently to large language models (3, 4). Large Language Models (LLMs) are transformer-based systems trained on vast amounts of text, which is broken down into smaller linguistic units known as tokens. They learn the relationships among these tokens using attention mechanisms, thereby capturing context and meaning (5), which are then stored as knowledge within the model's billions of parameters.

Current LLMs have many potential applications in dental medicine, such as clinical decision-support, radiology interpretation, dental education, and research methodology (4). They can read and summarize patient notes, highlight important findings, and organize information into a consistent format for electronic records (6). In education, they can explain clinical procedures or treatment options in plain language and provide quick answers to students' or clinicians' questions. In decision-making systems, they can compare a patient's information with known patterns from the literature to suggest possible diagnoses or management plans Sallam et al. (7). The promise of LLM and its potential in these applications are well documented in other health professions. However, despite its versatility, LLM outputs are limited by the information in their training data, which may be too general or incomplete for knowledge-intensive tasks (8) or outdated. Moreover, when dealing with specific domains or highly specialized queries, LLMs may generate hallucinations (i.e., plausible sounding but incorrect or fabricated information) (9, 10). In clinical contexts, LLMs should express uncertainty, such as stating “I don't know” rather than generating potentially misleading information due to hallucination. Fine tuning an LLM, or customizing an application of LLM through specified training, is a solution to prevent hallucination (11). For example, LLMs are trained or fine tuned on extensive biomedical text corpora, leading to the development of domain-specific systems such as BioBERT (12), ClinicalBERT (13), and BioGPT Luo et al. (14).

These specialized models contain knowledge-specific information, making text generation more accurate and better at referring to factual knowledge. However, the knowledge of these fine tuned LLMs will become outdated over time, and updating them is inefficient due to the large model parameters.

To minimize these limitations, Augmented Retrieval Generation (RAG) offers a promising framework that enhances LLM performance, ensuring precision, transparency, and contextual relevance in dental research and clinical applications. RAG systems work by integrating information retrieval mechanisms with generative models (8). The retrieval component searches external databases or knowledge sources, such as research articles, clinical guidelines, or patient records, to find the most relevant information for a given query. Then, the generative component, typically an LLM, uses the retrieved content to compose a coherent, contextually accurate response. By combining these two steps, RAG systems ground outputs in trusted sources of knowledge and reduce unsupported claims.

This architecture addresses these limitations by incorporating external knowledge sources that can be updated without retraining the model. In dentistry, where clinical recommendations and diagnostic criteria must be reproducible and supported by authoritative sources, this stability is crucial. Updating the underlying dental knowledge base allows RAG systems to incorporate new guidelines and research findings immediately, ensuring that outputs remain accurate, verifiable, and aligned with current standards. This mini-review summarizes the foundations of RAG, its emerging applications in dental research, and opportunities for future research in building trustworthy, domain-aware AI for dental research.

## RAG fundamentals

RAG effectively combines the parameterized knowledge of LLMs with non-parameterized external, often user defined, knowledge bases. The distinction between a model's parametric knowledge and its external non-parametric knowledge base is fundamental for understanding how RAG systems ground their outputs. Parametric knowledge is acquired through training LLMs and stored in neural network weights, representing a model's understanding and generalization of the training data, forming the foundation for generated responses. Non-parametric knowledge, on the other hand, resides in external sources such as vector databases; it is not encoded directly into the model but rather serves as updatable supplementary information. Non-parametric knowledge enables LLMs to access and leverage the most recent or domain- specific information, improving the accuracy and relevance of responses. Since model parameters cannot be updated dynamically, parametric knowledge can become outdated over time. Updating this knowledge typically requires fine tuning or retraining, which is not only computationally expensive but can also inadvertently alter the model's learned behavior, reducing the stability and reproducibility of its outputs. To overcome the limitations of purely parameterized models, language models can adopt a semi-parameterized approach by integrating a non-parameterized corpus database with parameterized models. Taken together, this method is known as Retrieval Augmented Generation (RAG). RAG systems link two processes: retrieval and generation. First, the system retrieves relevant information from an external knowledge source, and then the language model uses that evidence to produce a grounded and contextually accurate response. In this way, RAG reduces unsupported claims and improves factual reliability compared to a stand-alone LLM. A typical RAG architecture includes four components: indexing, retrieval, generation, and control.

**Figure 1:**
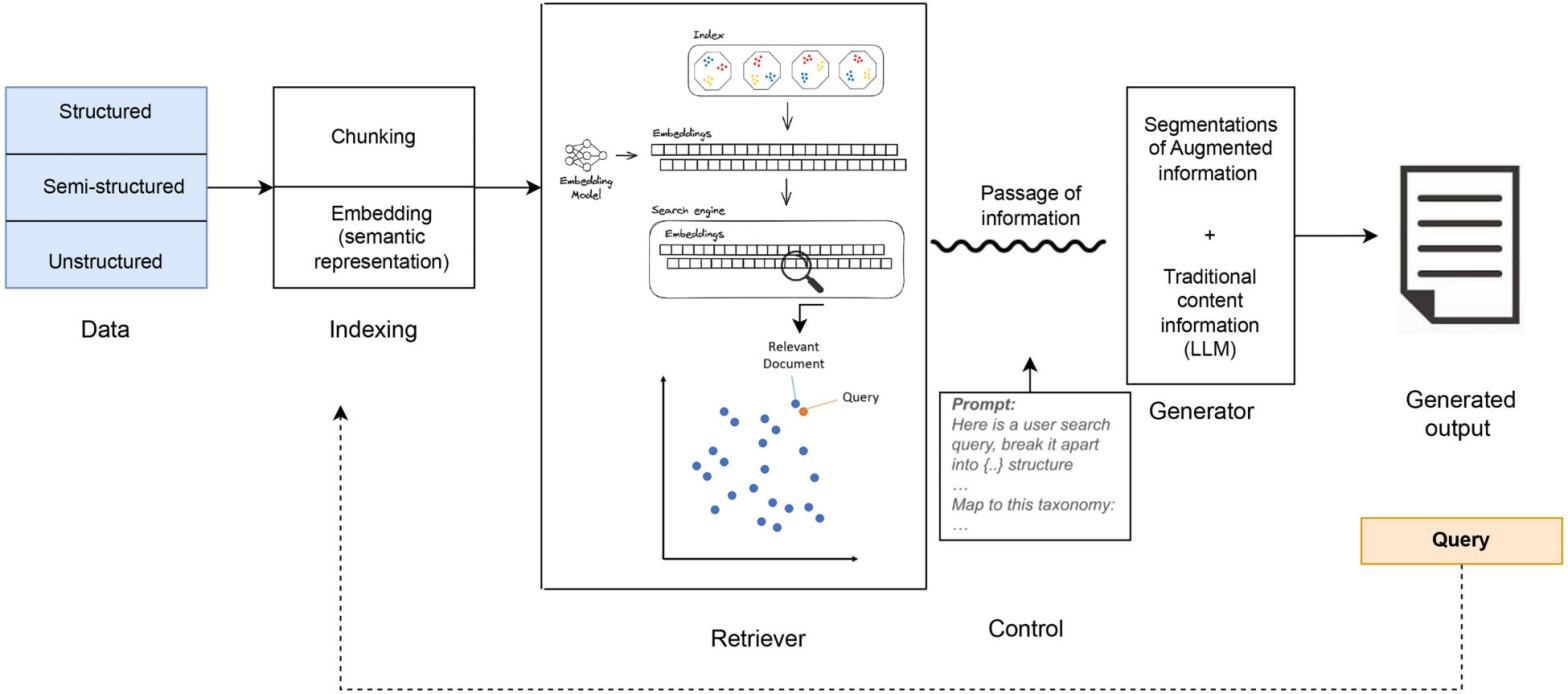
RAG pipeline.

The indexing layer is responsible for preparing the reference corpus. For applications in dentistry, this may include journal articles, clinical guidelines, patient notes, or radiographic reports. Longer documents are split into smaller units (“chunks”), and each chunk is converted into a numerical representation (“embedding”) (Figure 1). Embeddings capture semantic meaning, enabling the model to measure similarity between a question and the stored text based on concepts rather than exact keywords. Domain-specific embedding models may require fine tuning for dental terminology, as general models trained on open web data may not accurately capture specialized concepts. The retrieval component searches the indexed corpus to find the most relevant chunks for a given query. Several retrieval strategies exist. Sparse retrievers (e.g., BM25) match keywords, while dense retrievers (e.g., BERT-based models) match embeddings based on semantic similarity. Hybrid and re-ranker approaches combine both methods to maximize relevance. The quality of retrieval is critical because the generator can only use information that has been surfaced by this step (Figure 1). Different retrieval strategies also introduce distinct challenges in dental applications. Sparse retrievers such as BM25 depend on exact keyword matching and may fail to capture common dental synonyms or variations in terminology (e.g., “resorption,” “external cervical resorption,” or “root resorption”). Dense retrievers overcome lexical mismatch by relying on semantic embeddings, yet they may misrepresent rare or highly specialized dental terms if the embedding model is not trained on dentistry- specific corpora. Hybrid systems, which combine sparse and dense signals, can mitigate these limitations by improving recall while reducing retrieval noise, thereby offering more reliable performance when worki with heterogeneous or narrowly focused dental datasets (15).

The generator is an LLM that produces the final natural language answer. Instead of generating text only from its pre-trained parameters, the model is given the retrieved evidence and instructed to base its answer on that material. This ensures that responses are better aligned with current scientific knowledge, improves accuracy, and can even support explicit citation of source texts. Finally, the control layer manages the interaction between retrieval and generation. This includes prompt instructions, response format limitations, and the use of external tools (such as calculators or APIs). Control mechanisms are vital to ensure outputs are reliable, verifiable, and appropriate for clinical communication.

## The importance of RAG in dental research and application

LLMs have demonstrated remarkable progress in the biomedical sciences, leading to the development of domain-specific systems such as BioBERT (12), ClinicalBERT (13), and BioGPT (14). These specialized models are trained or fine tuned on extensive biomedical text corpora, allowing them to understand complex medical terminology and improve performance in clinical applications such as diagnosis, report generation, and literature synthesis. Dentistry had not seen comparable advances, but recent efforts are beginning to close this gap. Emerging systems such as the multimodal OralGPT-Omni model (16) demonstrate early attempts to adapt LLMs specifically for dental applications, and resources like DentalBench (17) now provide domain-specific corpora and benchmarks to support such adaptations. Despite these early developments, progress remains limited compared to medicine. The gap between medicine and dentistry is largely due to differences in data accessibility and digital infrastructure. Whereas medicine benefits from large open datasets and widely adopted, standardized EHR systems that facilitate model development and benchmarking, dental chart data are typically stored in private clinics, lack a consistent charting framework, and are fragmented across incompatible software systems. These data are rarely shared due to privacy regulations and the limited digitization of dental records (18). Although recent work has produced early fine-tuned dental LLMs, their development remains constrained by the small size, heterogeneity, and poor interoperability of available datasets. These structural barriers make large-scale fine-tuning difficult to sustain and nearly impossible to scale across institutions. Furthermore, the computational demands required for fine-tuning large LLMs exceed the technical capacity of most dental research environments, reinforcing the need for alternative approaches such as RAG.

RAG provides an efficient alternative to fine tuning by enabling models to access relevant information dynamically during inference. Rather than retraining an entire model, RAG can retrieve information from domain-specific sources, such as journal articles, clinical guidelines, or electronic health records, and integrate it into the model's reasoning. This approach not only reduces the computational cost but also allows continual updating of the knowledge base without retraining the core model. For instance, a RAG-enhanced system could draw on the most recent literature about implant materials or caries risk assessment and deliver evidence-based recommendations directly within a clinical workflow. In addition, RAG mitigates the domain bias that can occur when models trained primarily on medical or general biomedical text (e.g., ClinicalBERT) are applied to dental topics. By grounding responses in current, dentistry-specific information, RAG improves contextual relevance and accuracy. Therefore, RAG represents a practical and scalable methodology for both researchers and clinicians in dentistry, enabling evidence-based decision-making, educational support, and research synthesis without the high cost or data requirements of traditional fine tuning.

## RAG-based studies in dentistry

**Figure 2:**
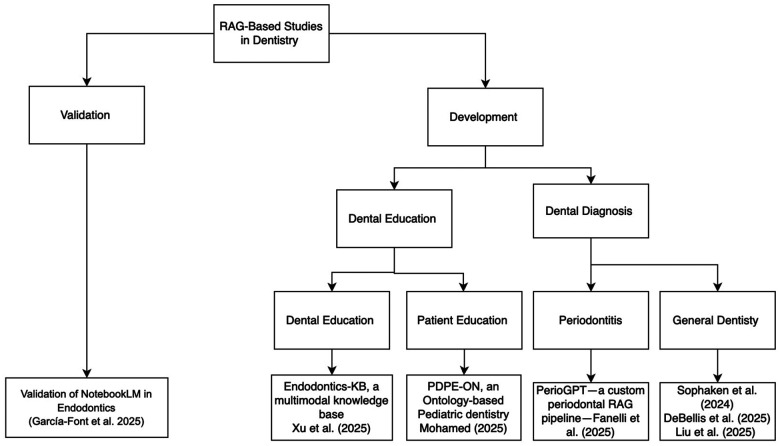
Summary of RAG-based studies in dentistry.

Studies included in this mini-review were identified through searches of PubMed, Scopus, and Google Scholar (January 2020–December 2025) using combinations of the terms “RAG,” “retrieval-augmented generation,” “generative language models,” or “LLM” with dental-related keywords such as “dental,” “dentistry,” “oral health,” and “oral medicine.” Articles were included if they (1) implemented a RAG architecture, (2) addressed applications in dental education, diagnosis, or patient communication, and (3) reported technical details or evaluation outcomes. Preprints were considered when peer-reviewed publications were unavailable, given the emerging nature of this research area. The review shows that RAG-based systems are being developed and validated for both educational and diagnostic applications, as summarized in Figure 2. The first validation of a RAG-enhanced system in dentistry was reported by Garcia-Font et al. (19). The study compared a traditional LLM (Google Gemini) with a retrieval augmented model (NotebookLM) in endodontics. The models were evaluated on 46 dichotomous questions concerning external cervical resorption. NotebookLM, which grounded its responses in curated literature rather than relying solely on pretrained knowledge, achieved an accuracy of 96%, outperforming Gemini's 89%. Although the higher accuracy of NotebookLM compared to Gemini is encouraging and represents an early step toward evidence-based validation of RAG models in dental contexts, the evaluation design has important limitations. The use of dichotomous (yes/no) questions restricts the ability to assess depth of reasoning, handling of uncertainty, and quality of justification, which are central to clinical decision-making. Future studies should employ open-ended, case-based scenarios and require explicit explanations to more rigorously evaluate how RAG systems support endodontic reasoning. Beyond validation, a growing body of work has focused on developing RAG systems tailored for dental education and clinical diagnosis. RAG-based systems are proving particularly valuable in dental education, where multimodal reasoning—combining textual, visual, and procedural information—is critical for training students. Xu et al. (20) introduced Endodontics-KB, a multimodal RAG knowledge base containing more than 2,200 text and video resources. Their model, EndoQ, integrated these resources using a retrieval mechanism that enabled LLMs to reference educational materials during question answering. Compared to general purpose LLMs such as GPT-4 and Qwen 2.5, EndoQ demonstrated higher scores in completeness, clinical reasoning, and professionalism. Completeness was assessed by examining whether responses covered all essential diagnostic elements relevant to each scenario. Clinical reasoning was measured by evaluating the logical coherence, justification, and sequencing of decision-making steps. Professionalism was assessed based on accuracy, tone, and adherence to clinical communication standards. Despite these promising findings, Endodontics-KB and EndoQ also present several constraints. Although the knowledge base is relatively large (over 2,200 text and video resources), the evaluation was conducted on a limited set of expert-designed questions and simulated scenarios, rather than real-world clinical cases. Moreover, constructs such as “completeness,” “clinical reasoning,” and “professionalism” were operationalized through expert ratings, which, while valuable, introduce subjectivity and may limit reproducibility across institutions. Larger, multi-center evaluations with standardized metrics would be needed to confirm the generalizability of these results.

Parallel work has explored RAG for patient education, emphasizing personalized, explainable communication. MOHAMED (21) developed PDPE-ON, an ontology-based knowledge framework for pediatric dentistry designed to support RAG-powered conversational agents. While the system was not directly linked to an LLM, its structured representation of fluoride guidance, eruption timelines, and age-specific care protocols provides a strong foundation for patient facing RAG applications that deliver consistent, evidence-based information in accessible language. A major advancement in diagnostic applications came with PerioGPT, a custom RAG pipeline for periodontal disease management developed by Fanelli et al. (22). Built on GPT-4o and a curated corpus of periodontal literature, PerioGPT demonstrated superior diagnostic accuracy and generative quality compared to five other dental chatbots. The retrieval component enabled dynamic integration of current research evidence and clinical guidelines, allowing the model to provide nuanced, case-specific recommendations. PerioGPT illustrates the potential of RAG for specialty decision support, but its scope is constrained by the composition of its underlying corpus. The model is optimized for periodontal content and may not generalize to broader restorative or multidisciplinary contexts, and the study provides limited detail on error types and failure modes. Without systematic reporting of dataset diversity, calibration across disease severities, and comparative performance on complex, ambiguous cases, it remains difficult to fully appraise the robustness of the system for routine clinical deployment. RAG approaches have also been extended to broader dental domains through several innovative frameworks. Sophaken et al. (23) implemented Graph-RAG, which integrates knowledge graphs and ontologies to organize unstructured dental literature into structured, concept based relationships. By linking related entities, such as materials, pathologies, and procedures, the model retrieves information based on semantic relations rather than mere text similarity. This structure aware design improves transparency and interpretability, which are crucial for treatment planning and diagnostic explanations.

Similarly, DeBellis et al. (24) developed an ontology-driven RAG system tailored for clinical use in India. By combining a dental materials ontology with LLM-based retrieval, their model provided explainable, context-specific guidance on restorative materials and clinical indications. The ontology constrained the model's reasoning, reducing hallucination and ensuring alignment with dental practice standards. Moreover, Liu et al. (25) introduced MedChain, a multi-agent system featuring a case-based RAG module (MedCase-RAG). Although developed for medicine, the framework's design which was focusing on retrieving comparable patient cases to guide sequential reasoning across referral, diagnosis, and treatment offers direct relevance to dentistry. Its emphasis on personalization, interactivity, and longitudinal case understanding aligns with the needs of dental diagnosis and treatment planning, particularly in specialties that involve radiographic interpretation and patient follow-up.

## Gaps and future opportunities for RAG in dentistry

Although the initial body of RAG-based research in dentistry has shown promising results, the field remains at a nascent and uneven stage of development. The reviewed studies vary widely in scope, methodology, and evaluation metrics, making it difficult to compare performance across systems. Some studies report accuracy on dichotomous endodontic questions (19), others use qualitative expert ratings (7), and still others employ specialty examination items or custom scoring criteria, such as the PerioGPT study which evaluated performance using specialist-written periodontal questions, items from the American Academy of Periodontology In-Service Examination, repeated-question reproducibility, and expert-rated complexity and accuracy scores (22). The absence of standardized evaluation frameworks limits the ability to benchmark RAG performance, assess clinical reliability, or determine readiness for real-world implementation. Developing shared, dentistry-specific evaluation metrics that are aligned with diagnostic accuracy, clinical reasoning quality, explainability, and patient safety represents a critical next step for advancing the field.

A recurrent theme across existing studies is RAG's ability to enhance factual grounding, particularly in high-risk decision-making scenarios such as endodontic pathology and periodontal diagnosis. Unlike general-purpose LLMs, which frequently hallucinate when encountering sparse or ambiguous dental information, retrieval grounding constraints models to validated sources, including incorrectly identifying resorptive lesions (19) or misclassifying periodontal status (22). Future research should investigate how RAG can be optimized to support complex diagnostic reasoning and synthesize multimodal evidence in these high-stakes contexts.

Another insight emerging from the reviewed work is the importance of structured retrieval, especially through ontologies and knowledge graphs (22). Systems such as Graph-RAG (5) and ontology-augmented retrieval pipelines (21) demonstrate that structured knowledge improves interpretability, reduces hallucinations, and aligns retrieved information with clinical reasoning patterns. Because dentistry involves many hierarchical relationships (e.g., tooth anatomy, material indications, caries staging, eruption sequences), structured retrieval frameworks offer a promising approach to building transparent, clinician trustworthy AI systems. Developing comprehensive dental knowledge graphs that potentially linked to SNODENT, ICD-DA, or new specialty ontologies should be considered a priority area for future development.

Furthermore, dentistry is inherently multimodal, with many decisions depending on radiographs, CBCT scans, photographs, diagrams, and procedural videos. The success of Endodontics-KB underscores the critical role of multimodal RAG plays in procedural disciplines such as endodontics, radiology, and oral surgery, where textual descriptions alone are insufficient. However, most existing RAG systems in dentistry rely solely on text retrieval. Future work should integrate visual embeddings, radiographic databases, and video-based procedural content to support richer, more clinically aligned decision-making.

Finally, patient-facing applications represent another area of opportunity. Ontology-driven frameworks such as PDPE-ON illustrate how RAG could deliver tailored, age-appropriate, and context-sensitive oral health information. Because oral health literacy varies widely across patient populations, RAG enables dynamic adjustment of explanations to a patient's developmental stage, reading level, and clinical scenario. While LLM applications currently dominate public attention, their reliable use in dental research and clinical practice remains limited due to concerns about hallucination, outdated knowledge, and inconsistent reasoning. RAG offers a more dependable alternative by grounding outputs in curated evidence, yet its implementation in real clinical environments presents several practical challenges. Real-time retrieval and re-ranking can impose significant computational demands, and maintaining a high-quality corpus requires continuous curation, version control, and governance to prevent outdated or contradictory information from influencing recommendations. The integration of patient-derived data also introduces legal and ethical considerations related to privacy, secure storage of vector embeddings, and safeguards against unintended data leakage. Moreover, clinical deployment will require rigorous quality assurance procedures, such as audit trails, transparent citation of sources, periodic validation, and clinician-in-the-loop oversight to ensure safety and reliability. Looking forward, progress will require addressing persistent issues such as domain shift, retrieval noise, small corpus sizes, and the lack of curated dental datasets. Purpose-built dental knowledge bases, specialty-specific corpora, and standardized evaluation tools will be essential for developing reliable, scalable RAG systems suited for clinical use. As the field matures, RAG has the potential to become a foundational technology for dental diagnostics, education, and patient communication.
